# Tetradecylthioacetic acid inhibits proliferation of human SW620 colon cancer cells - gene expression profiling implies endoplasmic reticulum stress

**DOI:** 10.1186/1476-511X-10-190

**Published:** 2011-10-25

**Authors:** Anne G Lundemo, Caroline HH Pettersen, Kjetil Berge, Rolf K Berge, Svanhild A Schønberg

**Affiliations:** 1 Norwegian University of Science and Technology, Faculty of Medicine, Department of Laboratory Medicine, Children's and Women's Health, PO Box 8905, N-7491 Trondheim, Norway; 2 Institute of Medicine, Section of Medical Biochemistry, Haukeland University Hospital, University of Bergen, N-5021 Bergen, Norway

**Keywords:** Cell cycle, Colon cancer, Docosahexaenoic acid, Endoplasmic reticulum stress, Gene expression analysis, Phosphorylated eIF2α, Tetradecylthioacetic acid, Thia fatty acids, Unfolded protein response

## Abstract

**Background:**

Previous reports have shown an antiproliferative effect of the synthetic, 3-thia fatty acid tetradecylthioacetic acid (TTA) on different cancer cells *in vitro *and *in vivo*. The mechanisms behind the observed effects are poorly understood. We therefore wanted to explore the molecular mechanisms involved in TTA-induced growth inhibition of the human colon cancer cell line SW620 by gene expression profiling.

**Methods:**

An antiproliferative effect of TTA on SW620 cells *in vitro *was displayed in real time using the xCELLigence System (Roche). Affymetrix gene expression profiling was performed to elucidate the molecular mechanisms behind the antiproliferative effect of TTA. Changes in gene expression were verified at protein level by western blotting.

**Results:**

TTA reduced SW620 cell growth, measured as baseline cell index, by 35% and 55% after 48 h and 72 h, respectively. We show for the first time that TTA induces an endoplasmic reticulum (ER) stress response in cancer cells. Gene expression analysis revealed changes related to ER stress and unfolded protein response (UPR). This was verified at protein level by phosphorylation of eukaryote translation initiation factor 2 alpha (eIF2α) and downstream up-regulation of activating transcription factor 4 (ATF4). Transcripts for positive and negative cell cycle regulators were down- and up-regulated, respectively. This, together with a down-regulation of Cyclin D1 at protein level, indicates inhibition of cell cycle progression. TTA also affected transcripts involved in calcium homeostasis. Moreover, mRNA and protein level of the ER stress inducible C/EBP-homologous protein (CHOP), Tribbles homolog 3 (Drosophila) (TRIB3) and CCAAT/enhancer binding protein beta (C/EBPβ) were enhanced, and the C/EBPβ LIP/LAP ratio was significantly increased. These results indicate prolonged ER stress and a possible link to induction of cell death.

**Conclusion:**

We find that TTA-induced growth inhibition of SW620 cells seems to be mediated through induction of ER stress and activation of the UPR pathway.

## Background

Colon cancer is one of the most common incident cancers across Europe [1]. There is an urgent need for finding novel, alternative or supplementary cancer treatments. Polyunsaturated fatty acids (PUFAs) are able to inhibit growth of both colon and several other types of cancer cells *in vitro *and *in vivo *([2-5] and reviewed in [6-8]). During the last two decades, chemical modified fatty acid (FA) analogs have been produced in an attempt to achieve FAs having increased metabolic stability and more selective and targeted effects (reviewed in [9]). Among these is the bioactive, saturated 3-thia FA tetradecylthioacetic acid (TTA). TTA has been shown to have both a cardioprotective effect, as well as an antiproliferative effect on cancer cells (reviewed in [10]). TTA has been found to inhibit growth of glioma [11,12], leukemia [13-16] and colon cancer cell lines [11,17]* in vitro *and *in vivo*, and hepatoma [18] and breast cancer cells [19]* in vitro*. TTA seems to be more potent in reducing cell growth compared with the omega-3 (n-3) PUFAs eicosapentaenoic acid (EPA) and docosahexaenoic acid (DHA) [20]. A diet containing TTA has also resulted in increased vascularisation of colon cancer xenografts in mice [17] and improved the survival of mice having leukemia xenografts [11,14]. TTA food supplementation seems to be generally well tolerated by healthy persons [15,21], and TTA therefore may have a potential in cancer treatment alone or in combination with other therapies.

The structure of TTA is equal to the saturated palmitic acid (PA), except that TTA has a sulphur atom inserted at the third position in the carbon chain [19]. The sulphur atom makes TTA resistant to mitochondrial β-oxidation and probably contributes substantial to its biological effects (reviewed in [10]). TTA is capable of reducing the growth of cancer cells that are not growth inhibited by PA [17,20,22]. TTA is degraded relatively slowly to various dicarboxylic acids via ω-oxidation and sulphur oxidation in the endoplasmic reticulum (ER) and subsequent β-oxidation in the peroxisomes. Except from blocked mitochondrial β-oxidation, the chemical properties and metabolism of TTA resembles those of normal FAs. TTA is activated by binding to coenzyme A and incorporated into various lipids, especially phospholipids (reviewed in [10,23]).

Before any recommendations regarding use of TTA in cancer treatment can be given, it's important to elucidate the molecular mechanisms underlying the growth inhibitory effect of TTA. In some, but not all, cancer cells, TTA inhibits cancer cell growth via increased lipid peroxidation and oxidative stress [12], or partly via activation of peroxisome proliferator activated receptor gamma [22]. Also, TTA has been shown to induce apoptosis in several glioma [12,13] and leukemia cell lines [13,14]. Induction of apoptosis seems to be related to effects on mitochondria. TTA can induce a decrease in mitochondrial membrane potential [13,24] and lead to release of cytochrome C (cyt C) and a reduction in mitochondrial glutathione, the latter indicating a selective modulation of the mitochondrial redox equilibrium [13]. Most of the biological effects of TTA assumed to be implicated in mediating the inhibitory effect of TTA on cancer cells do not seem to be specific for TTA, since they also are assumed to be involved in the growth inhibitory effect of other FAs like n-3 PUFAs [25-27].

We have previously shown that TTA inhibits the growth of SW620 human colon cancer cells *in vitro *and *in vivo *[17]. SW620 cell growth is also inhibited by n-3 PUFAs [2,4]. By using gene expression analysis, we found that DHA induces extensive changes in the expression of transcripts involved in biological pathways like ER stress and unfolded protein response (UPR), protein degradation, Ca^2+ ^homeostasis, cell cycle progression and apoptosis [28]. Others have found that PA also can induce ER stress in human [29] and rat cancer cells [30].

The main functions of ER are protein synthesis and folding, lipid synthesis and maintenance of Ca^2+ ^homeostasis. Disruption of these processes causes accumulation of misfolded proteins in ER lumen, leading to ER stress and activation of the cellular stress response UPR. The purpose of UPR is to restore cell homeostasis and promote cell survival, but during prolonged ER stress, apoptosis may be activated. During ER stress, one of the three ER stress sensors; eukaryotic translation initiation factor 2 alpha (eIF2α) kinase 3 (EIF2AK3/PERK) is known to phosphorylate eIF2α, thereby attenuating global protein synthesis to reduce the protein load of ER. Reduced synthesis of e.g. cyclin D1 promotes cell cycle arrest, creating time to cope with the stress. However, translation of certain mRNAs is allowed, like mRNAs for activating transcription factor 4 (ATF4) and its downstream target C/EBP-homologous protein (CHOP) [31,32]. CHOP, which is also regulated by X-box binding protein 1 (XBP-1) (reviewed in [33]), promotes apoptosis by down-regulating anti-apoptotic factors like B-cell lymphoma 2 (Bcl-2) [34] and up-regulating pro-apoptotic factors like the Bcl-2-interacting protein Bim [35].

During ER stress XBP-1 also activates the transcription factor CCAAT/enhancer binding protein beta (C/EBPβ) [36], which participates in regulation of differentiation, cell growth, cell survival and apoptosis [37,38]. Human C/EBPβ is mainly expressed as two 46 and 42 kDa liver enriched activator protein isoforms (LAP and LAP*) and a 20 kDa liver enriched inhibitor protein isoform (LIP) [39], which are synthesized from different AUG start codons within the C/EBPβ mRNA [40]. Since LIP lacks a transcription activation domain [41] and suppresses the transactivation by LAP, the LIP/LAP ratio influences on the extent of C/EBPβ-activated transcription [40].

LIP has been shown to decrease during early ER stress, and increase during prolonged ER stress, causing an increase in the LIP/LAP ratio [42]. Increased LIP seems to be essential for CHOP-induced apoptosis, since heterodimerization of LIP with CHOP promotes nuclear translocation of CHOP and pro-apoptotic gene regulation by CHOP [43]. In addition, CHOP-C/EBPβ [44] and CHOP-ATF4 [45] heterodimeres up-regulate the pro-apoptotic pseudo-kinase Tribbles homolog 3 (Drosophila) (TRIB3), which can promote apoptosis by inhibiting the anti-apoptotic kinase Akt [46] and the transcription factor nuclear factor kappa-light-chain-enhancer of activated B cells (NF-κB) [47]. CHOP- and TRIB3-promoted cell death might be accompanied by the ER stress-induced caspase 4 (CASP4) and calpains [32,48,49].

In this report we have used Affymetrix gene expression analysis to search for possible molecular mechanisms underlying the growth inhibitory effect of TTA on SW620 cells *in vitro*. The results indicate that TTA reduces SW620 cell growth through activation of ER stress and UPR, by a similar mechanism as observed after treatment with DHA [28].

## Results

### TTA reduces growth of SW620 cells

**Figure 1:**
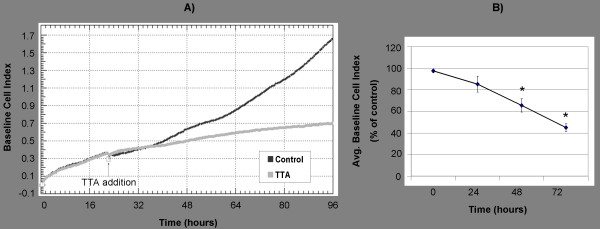
**Real time monitoring of TTA-induced growth inhibition of SW620 cells**. SW620 colon cancer cells were seeded in 16 well E-plates. After ~24 h, TTA (75 μM) or NaOH (control) were added. Cell growth was monitored using the xCELLigence RTCA DP Instrument (Roche). (A) SW620 cell growth presented as baseline cell index from one representative experiment. (B) Average baseline cell index (± SD) of TTA-treated cells, presented as percent of control, at indicated time points. Mean was calculated from at least two duplicate measurements in four independent experiments. * Significantly different from control (one-tailed Student's t-test, P < 0.05).

In accordance with previous results [17], we verified a growth inhibitory effect of TTA (75 μM) on SW620 colon cancer cells by cell counting (data not shown) and by measuring the growth real time by using the xCELLigence System (Roche) (Figure 1A). The SW620 growth curve, which is presented as baseline cell index, showed that TTA (75 μM) significantly reduced the baseline cell index by 35% and 55% after 48 h and 72 h, respectively, compared to the control (Figure 1B).

### TTA induces extensive changes at mRNA level indicating ER stress and UPR

In an attempt to unravel the mechanisms underlying the growth-inhibiting effect of TTA, SW620 cells were cultivated in the absence or presence of 75 μM TTA for 24 h, followed by gene expression profiling. Differentially expressed transcripts are presented and listed by full and short names in Table 1 and Additional file 1.

**Table 1:** Gene expression results

Gene Symbol	Affymetrix ID	Refseq NCBI ID	Transcript name	SW620 Fold Change
				
				24 h
**ER stress and unfolded protein response**				

ATF3	202672_s_at	NM_001030287 NM_001040619 NM_001674 NM_004024	Activating transcription factor 3	3.5

ATF4	200779_at	NM_001675 NM_182810	Activating transcription factor 4	1.7

ATF6	217550_at 231927_at 203952_at	NM_007348	Activating transcription factor 6	1.5 1.3 1.2

CEBPB	212501_at	NM_005194	CCAAT/enhancer binding protein (C/EBP), beta	3.2

CHOP/DDIT3	209383_at	NM_001130101 NM_001130102 NM_004083 NM_005693	DNA-damage-inducible transcript 3	2.7

GADD34	37028_at 202014_at	NM_014330	Growth arrest and DNA-damage-inducible 34	2.0 2.0

IRE1/ERN1	235745_at	NM_001433	Inositol-requiring enzyme 1/Endoplasmic reticulum to nucleus signaling 1	1.7

NRF2/NFE2L2	201146_at	NM_001145412 NM_001145413 NM_006164	Nuclear factor (erythroid-derived 2)-like 2	1.3

TRIB3	1555788_a_at 218145_at	NM_021158	Tribbles homolog 3 (Drosophila)	5.1 4.9

XBP1	242021_at 200670_at	NM_001079539 NM_005080	X-box binding protein 1	1.2 1.6

**Chaperones/Protein folding/Unfolded protein response/Protein degradation/Amino acid synthesis**				

ASNS	205047_s_at	NM_001673 NM_133436 NM_183356	Asparagine synthetase	4.8

CREB3L2	212345_s_at	NM_194071	cAMP responsive element binding protein 3-like 2	1.4

CREB3L3	234361_at	NM_032607	cAMP responsive element binding protein 3-like 3	3.0

DNAJB2	202500_at	NM_001039550 NM_006736	DnaJ (Hsp40) homolog, subfamily B, member B2	1.3

DNAJB14	222850_s_at	NM_001031723	DnaJ (Hsp40) homolog, subfamily B, member B14	1.2

DNAJC24	242562_at	NM_181706	DnaJ (Hsp40) homolog, subfamily C, member 24	1.4

EDEM1	203279_at	NM_014674	ER degradation enhancer, mannosidase alpha-like 1	1.3

EDEM3	220342_x_at	NM_025191	ER degradation enhancer, mannosidase alpha-like 3	1.2

ERO1LB	231944_at	NM_019891	ERO1-like beta (S. cerevisiae)	1.3

HMOX1/HSP32	203665_at	NM_002133	Heme oxygenase (decycling) 1	1.6

HSPA13	202557_at 202558_s_at	NM_006948	Heat shock 70kDa protein 13	1.4 1.7

PDIA2	206691_s_at	NM_006849	Protein disulfide isomerase family A, member 2	1.2

PFDN2	218336_at	NM_012394	Prefoldin 2	1.4

PSMB1	214289_at	NM_002793	Proteasome subunit, beta type, 1	1.3

SQSTM1	201471_s_at 213112_s_at 239004_at 244804_at	NM_001142298 NM_001142299 NM_003900	Sequestosome 1	2.1 3.1 1.3 2.1

UBE2B	239163_at	NM_003337	Ubiquitin-conjugating enzyme E2B (RAD6 homolog)	1.5

SEC61A2	219499_at 228747_at	NM_001142627 NM_001142628 NM_018144	Sec61 alpha 2 subunit (S. cerevisiae)	1.3 1.2

SEC61B	244700_at	NM_006808	Sec61 beta subunit	1.6

SEC63	201914_s_at 201915_s_at 201916_s_at	NM_007214	SEC63 homolog (S. cerevisiae)	1.3 1.2 1.3

**Ca2^+ ^homeostasis, signaling and transport**				

ATP2B4/PMCA4	205410_s_at 212135_s_at 212136_at	NM_001001396 NM_001684	ATPase, Ca^2+ ^transporting, plasma membrane 4	1.5 2.7 5.4

CAPN1	232012_at	NM_005186	Calpain 1, (mu/I) large subunit	1.4

CAPN2	208683_at 214888_at	NM_001146068 NM_001748	Calpain 2, large subunit	1.4 1.8

CAPN5	205166_at	NM_004055	Calpain 5	1.4

HERPUD1	217168_s_at	NM_001010989 NM_001010990 NM_014685	Homocysteine-inducible, endoplasmic reticulum stress-inducible, ubiquitin-like domain member 1	1.9

ITPR1	244090_at	---	Inositol 1,4,5-triphosphate receptor, type 1	1.3

ITPR3	201187_s_at 201188_s_at 201189_s_at	NM_002224	Inositol 1,4,5-triphosphate receptor, type 3	1.3 1.5 1.5

PLCD3	234971_x_at 1552476_s_at	NM_133373	Phospholipase C, delta 3	1.3 1.3

S100A10	200872_at 238909_at	NM_002966	S100 calcium binding protein A10	1.2 2.4

S100P	204351_at	NM_005980	S100 calcium binding protein P	4.9

STC2	203438_at 203439_s_at	NM_003714	Stanniocalcin 2	2.1 2.7

**Cell cycle/Apoptosis**				

ATF5	204998_s_at 204999_s_at	NM_012068	Activating transcription factor 5	1.5 1.7

AURKA	208080_at	NM_003600 NM_198433 NM_198434 NM_198435 NM_198436 NM_198437	Aurora kinase A	-1.3

BIRC5	202094_at 202095_s_at 210334_x_at	NM_001012270 NM_001012271 NM_001168	Baculoviral IAP repeat-containing 5/Survivin	-1.7 -1.3 -1.3

CASP4	213596_at	NM_001225 NM_033306	Caspase 4	1.4

CCNA2	203418_at 213226_at	NM_001237	Cyclin A2	-1.2 -1.2

CCND3	201700_at	NM_001136017 NM_001136125 NM_001136126 NM_001760	Cyclin D3	-2.0

CCNE1	213523_at	NM_001238 NM_057182	Cyclin E1	-1.3

CCNE2	205034_at 211814_s_at	NM_057749	Cyclin E2	-2.9 -3.1

CCNF	204827_s_at	NM_001761	Cyclin F	-1.2

CDC2/CDK1	203214_x_at	NM_001130829 NM_001786 NM_033379	Cell division cycle 2, G1 to S and G2 to M	-1.2

CDK2	204252_at 211804_s_at	NM_001798 NM_052827	Cyclin-dependent kinase 2	-1.3 -1.4

CDK4	202246_s_at	NM_000075	Cyclin-dependent kinase 4	-1.4

CDK5	204247_s_at	NM_001164410 NM_004935	Cyclin-dependent kinase 5	-1.5

CDK6	224847_at 224848_at 224851_at 243000_at	NM_001145306 NM_001259	Cyclin-dependent kinase 6	-1.4 -1.5 -1.3 -1.3

CDKN2A	211156_at	NM_000077 NM_058195 NM_058197	Cyclin-dependent kinase inhibitor 2A (melanoma, p16, inhibits CDK4)	1.3

CDKN2B	207530_s_at 236313_at	NM_004936 NM_078487	Cyclin-dependent kinase inhibitor 2B (p15, inhibits CDK4)	1.5 2.0

CDKN2D	210240_s_at	NM_001800 NM_079421	Cyclin-dependent kinase inhibitor 2D (p19, inhibits CDK4)	1.2

CUL1	238509_at	NM_003592	Cullin 1	1.4

KLF4	220266_s_at 221841_s_at	NM_004235	Kruppel-like factor 4 (gut)	1.9 2.4

PDCD4	202731_at 212593_s_at	NM_014456 NM_145341	Programmed cell death 4 (neoplastic transformation inhibitor)	1.3 1.3

PDCD6	222152_at 222380_s_at	NM_013232	Programmed cell death 6	1.3 1.3

SFN	33322_i_at 33323_r_at 209260_at	NM_006142	Stratifin	1.6 1.7 1.6

Significantly differentially expressed transcripts in SW620 treated with TTA (75 μM) for 24 h as determined by Affymetrix microarray analysis (P < 0.05).

**Figure 2:**
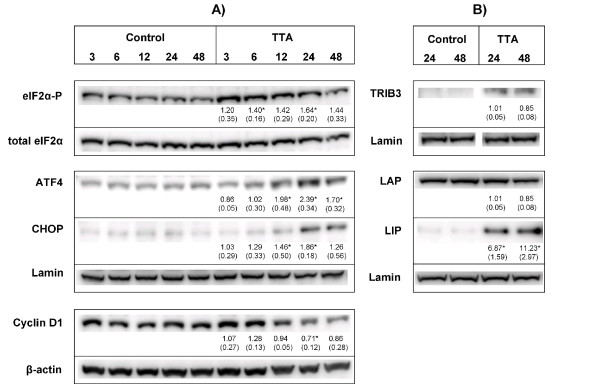
**TTA induces proteins involved in ER stress and UPR**. SW620 cells were treated with TTA (75 μM) or NaOH (control) for indicated time periods, and proteins were quantified by western blotting. (A) Analysis of eIF2α-P and Cyclin D1 from cytosolic protein extracts and ATF4 and CHOP from nuclear protein extracts. (B) Analysis of TRIB3 and the C/EBPβ protein isoforms LAP (45 kDa) and LIP (20 kDa) from nuclear protein extracts. Blots were quantified, and band intensities normalized relative to the respective loading control; β-actin (Cyclin D1), Lamin (nuclear extracts) or total level of eIF2α (eIF2α-P), to adjust for unequal protein loading within the membranes. Band intensities were related to the 24 h control band to adjust for differences in signal intensities between the membranes, except for TRIB3 where the band intensities are related to the 24 h TTA band (not expressed in control). Quantified results show mean fold change (± SD) of TTA-samples relative to control at indicated time periods for three independent experiments. One representative blot is shown. * Significantly different from control (one-tailed Student's t-test, P < 0.05).

After incubation with TTA, the transcription level of several genes involved in ER stress and UPR were affected. The ER stress sensors inositol-requiring enzyme 1 (IRE1) and activating transcription factor 6 (ATF6), and downstream targets of these, like XBP-1, were up-regulated (Table 1). However, most changes in the ER stress signaling pathway were found downstream of PERK. Phosphorylation of eIF2α by PERK is a hallmark of ER stress (reviewed in [31]). After TTA treatment, phosphorylated eIF2α (eIF2α-P) was found significantly increased after 6 h and 24 h (Figure 2A).

Phosphorylation of eIF2α induces transcription of ATF4 and its downstream targets, like ATF3, CHOP, growth arrest and DNA-damage-inducible 34 (GADD34), TRIB3 [50,51] and heme oxygenase 1 (HMOX1) [52]. All these transcripts were up-regulated upon TTA treatment (Table 1). Moreover, the protein levels of ATF4, CHOP (Figure 2A) and TRIB3 (Figure 2B) were also increased. ATF4 is known to induce genes involved in amino acid synthesis, and several transcripts involved in amino acid synthesis and transport were also up-regulated (Table 1 and Additional file 1).

When UPR is activated, cells try to up-regulate the folding capacity e.g. through up-regulation of chaperones and heat shock proteins (Hsps) [31]. Transcripts of the folding machinery were found to be up-regulated in the SW620 cells after incubation with TTA. These include prefoldin 2 (PFDN2), DnaJ (HSP40) homolog, subfamily B, member 2 (DNAJB2), DNAJB14, DnaJ (Hsp40) homolog, subfamily C, member 24 (DNAJC24), heat shock protein 13 (HSPA13), HSP32 (HMOX1) and hypoxia up-regulated 1 (HYOU1) (Table 1 and Additional file 1). Protein disulfide isomerase family A, member 2 (PDIA2) and ERO1-like beta (ERO1LB), which are known to catalyze protein folding [31,53], were also up-regulated (Table 1). However, some DnaJ (Hsp40) homolog subfamily members and Hsps were also found to be down-regulated (Additional file 1).

During ER stress, improperly folded proteins are transferred to ER degradation enhancer, mannosidase alpha-like protein (EDEM) and translocated to cytosol for proteasomal degradation; a process called ER associated degradation (ERAD) [31]. EDEM1 and EDEM3, in addition to several proteasomal subunits and ubiquitin-related transcripts, were up-regulated upon TTA treatment. Also, proteasome subunit, beta type, 1 (PSMB1), ubiquitin-conjugating enzyme E2B (RAD6 homolog) (UBE2B) and cullin1 (CUL1) were up-regulated (Table 1 and Additional file 1). In addition, TTA also up-regulated sequestosome 1 (SQSTM1), which is able to sequester and shuttle polyubiquitinated, misfolded proteins to the proteasome (reviewed in [54]) (Table 1). Moreover, TTA up-regulated transcripts for parts of the ER translocation machinery which transports proteins to the cytosol, like Sec61 alpha 2 subunit (SEC61A2), Sec61 beta subunit (SEC61B) and SEC63 homolog (SEC63) (Table 1).

### TTA affects Ca^2+ ^homeostasis

Another indication of an ER stress condition in SW620 cells treated with TTA is the altered expression of transcripts coding for proteins involved in Ca^2+ ^homeostasis. The more than 5-fold up-regulation of ATPase, Ca^2+^-transporting, plasma membrane 4 (ATP2B4/PMCA4) might indicate the presence of a high Ca^2+ ^concentration in the cytosol (Table 1). A possible high Ca^2+ ^level in the cytosol may be part of the ER stress response, with Ca^2+ ^leaking from ER through the up-regulated IP3 receptors, type 1 and 3 (ITPR1 and 3, Table 1). We also found Phospholipase C D3 (PLCD3), which is known to participate in the generation of IP3 [55], to be up-regulated at mRNA level after TTA-treatment (Table 1).

Among other important Ca^2+^-related transcripts, we found several S100 Ca^2+ ^binding proteins to be differentially expressed after TTA-treatment, e.g. S100P and S100A10 were up-regulated (Table 1 and Additional file 1). Also, TTA caused up-regulation of Stanniocalcin 2 (STC2), which is induced downstream of PERK and critical for survival after UPR [56]. TTA further up-regulated homocysteine-inducible endoplasmic reticulum stress-inducible ubiquitin-like domain member 1 (HERPUD1) (Table 1), which is known to counteract Ca^2+ ^disturbances during ER stress [57].

### Effect of TTA on cell cycle

TTA affected the mRNA level of several cell cycle transcripts, most of them having rather small fold change values. Positive cell cycle regulators were down-regulated and negative regulators were up-regulated, as outlined below.

The cell cycle is known to be positively regulated by cyclins (CCNs) and cyclin dependent kinases (CDKs). The mRNA level of cyclin D3 (CCND3), but not cyclin D1, was down-regulated upon TTA treatment (Table 1). However, the protein level of cyclin D1 was significantly decreased after incubation with TTA (Figure 2A). Moreover, transcripts for CCNA2, CCNE1, CCNE2, CCNF, CDK1/CDC2, CDK2, CDK4, CDK5 and CDK6 were down-regulated (Table 1), indicating inhibition of cell cycle progression. The CDK4 inhibitors CDK inhibitor 2A (CDKN2A), CDKN2B and CKDN2D, which are negative regulators of cell cycle, were increased (Table 1). In addition, the tumor suppressor programmed cell death 4 (PDCD4), which is able to reduce the protein level of CDK4 [58], and PDCD6 were up-regulated (Table 1).

Other important cell cycle related transcripts that were up-regulated by TTA were ATF5, stratifin (SFN/14-3-3 sigma) and kruppel like factor 4 (KLF4) (Table 1). TTA also induced down-regulation of aurora kinase A (AURKA) and survivin/baculoviral IAP repeat-containing 5 (BIRC5) (Table 1).

### TTA changes expression of transcripts involved in ER stress-related apoptosis

Transcripts related to ER stress-induced apoptosis were also up-regulated upon TTA treatment. In addition to the up-regulation of CHOP and TRIB3, TTA also increased transcription of CASP4, calpain 1, large subunit (CAPN1), CAPN2 and CAPN5 (Table 1).

The C/EBPβ LIP isoform is known to augment ER stress-induced cell death by interfering with the expression of TRIB3 [59]. C/EBPβ (CEBPB) was up-regulated 3.2-fold at mRNA level (Table 1) after TTA treatment. Western blotting mainly displayed two C/EBPβ isoforms; a highly expressed 45 kDa LAP isoform and a lower expressed 20 kDa LIP isoform. The 42 kDa LAP* isoform was only found in very low amounts (Figure 2B). TTA did not affect the level of LAP, but induced a significant 6.9- and 11.2-fold induction of LIP after 24 and 48 h, respectively (Figure 2B). The accumulation of LIP at these time points caused a significant 6.6- and 13.3-fold increase in the LIP/LAP ratio after 24 h and 48 h, respectively, compared to control cells.

During ER stress, cAMP responsive element binding protein 3-like 2 and 3 (CREB3L2 and CREB3L3) are induced in order to attenuate ER stress-induced cell death [60]. These transcripts were also up-regulated (Additional file 1).

## Discussion

Several studies have shown that TTA inhibits the growth of various cancer cells *in vitro *and *in vivo *[13,20,22]. We have previously shown that TTA inhibits DNA synthesis in SW620 human colon cancer cells in a time- and dose-dependent manner [17], and the growth inhibitory effect of TTA on this cell line was confirmed in the current study by xCELLigence proliferation assay. This assay monitors cell growth real time by measuring changes in electric impedance between two golden electrodes embedded in the bottom of the cell culture wells. The impedance, which is converted to a cell index value, is directly proportional to the number of cells and also reflects the cells' viability, morphology and adhesion strength [61]. The baseline cell index declined compared to the control from approximately 20 hours after TTA-supplementation. Previous studies have indicated that TTA affects multiple biochemical pathways that might play a role in its growth-limiting effect on different cancer cells. However, the exact mechanisms are not yet clear.

This is the first study demonstrating that TTA is able to induce ER stress in cancer cells. Transcripts involved in ER stress and downstream of all three ER stress sensors, like ATF6, IRE1, XBP-1 and ATF4, were found to be differentially expressed after treating SW620 cells with TTA. Also, the increased expression of Hsps and DNAJs indicates that the cells try to increase their protein folding capacity. The up-regulation of EDEM1 and EDEM3, SQSTM1, proteasomal subunits and ubiquitin-related transcripts indicates activation of ERAD.

The PERK branch of UPR was found to be activated, supported by the increased protein level of eIF2α-P and ATF4, as well as up-regulation of several downstream targets of ATF4, like ATF3, ASNS, CHOP and TRIB3, at mRNA level. CHOP and TRIB3, also induced at protein level, are known to link ER stress to ER stress-induced cell death (reviewed in [32]). The enhanced level of these proteins and transcripts for CASP4 and calpains indicates that TTA causes prolonged ER stress and direct the cell fate towards induction of death. We have previously shown that DHA induces ER stress with increased eIF2α-P, ATF4 and CHOP in the same cell line [28,62]. We also found HL-60 leukemia cells to induce ER stress upon EPA treatment [63].

In SW620 cells, TTA up-regulated the C/EBPβ LIP isomer, while LAP was unchanged. LIP is capable of modulating the ER stress response by augmenting cell death, e.g. by enhancing the activity of CHOP [43]. On the contrary, LAP may attenuate ER stress and ER stress induced cell death by inhibiting CHOP [59,64]. However, Li *et al*. found that LAP modulated the ER stress response by enhancing the expression of pro-apoptotic genes. They also proposed that the ER stress response may be modulated by regulation of the LIP/LAP ratio [42]. We found this ratio to be increased after 24 and 48 h of TTA treatment, supporting the assumption that TTA causes prolonged ER stress and a switch towards induction of cell death. Even if C/EBPβ mRNA was up-regulated, we only found an increase of LIP at protein level. Different regulation of LIP and LAP might be explained by different synthesis and degradation rates of these isomers [42].

Our results also indicate that TTA affects several transcripts involved in cell cycle progression. Transcripts involved in regulation of both the G1/S and G2/M phases were differentially expressed. In general, positive cell cycle regulators (like CDKs, cyclins, AURKA and survivin) were down-regulated, and negative cell cycle regulators (like ATF5, SFN and KLF4) were up-regulated, which indicates a stop in the cell cycle progression. We have previously demonstrated that DHA treatment of the SW620 cells affects several cell cycle transcripts and proteins like CDKs, cyclins and survivin etc. [62] and arrests the cells in the G2/M phase of the cell cycle [2]. Brewer *et. al*. stated that cell cycle arrest might be induced during ER stress to prevent cells from completing cell division when the conditions are compromising proper folding and assembly of proteins [65].

TTA also seems to induce changes in expression of transcripts involved in maintenance of Ca^2+ ^homeostasis. The ER lumen contains a high Ca^2+ ^concentration, and induction of ER stress may either come from and/or culminate with a release of Ca^2+ ^from the ER (reviewed in [66,67]). ER stress-accompanied changes in the Ca^2+ ^metabolism may lead to a pro-apoptotic signal via the mitochondria [66]. We have previously shown that DHA and EPA affect Ca^2+ ^homeostasis in cancer cells [28,63]. Apoptosis has been observed in other cancer cells treated with TTA [13,22], and it has been proposed that this effect is mediated via mitochondrial alterations, such as decrease in mitochondrial membrane potential and release of Cyt C [13]. CHOP and C/EBPβ may be involved in the regulation of mitochondrial stress genes like TRIB3 [44] in response to accumulation of unfolded proteins in mitochondria [68]. Their up-regulation by TTA may indicate that the SW620 cells are undergoing a mitochondrial stress after TTA treatment.

Madsen *et al*. found TTA to be incorporated mainly into phospholipids in liver after TTA supplementation to rats [69]. The inner mitochondrial membrane phospholipid cardiolipin (CL) is essential for the function of several mitochondrial protein complexes. It interacts with mitochondrial Cyt C, which might utilize reactive oxygen species and cause oxidation of CL. Oxidized CL is required for release of pro-apoptotic factors like Cyt C to cytosol [70]. Enrichment of EPA and DHA in CL isolated from colonic mucosa in rats fed fish oil or EPA/DHA ethyl esters might result in high susceptibility to lipid peroxidation due to the FAs' high degree of unsaturation. This may trigger release of pro-apoptotic factors from mitochondria (reviewed in [71]). Decreased synthesis of CL has also been related to Cyt C release from mitochondria during PA-induced apoptosis (reviewed in [9]). It remains to be investigated if this is the case with TTA.

## Conclusions

TTA induces extensive changes in the expression of genes involved in several biological pathways. TTA causes accumulation of misfolded proteins in ER, causing ER stress and activation of UPR. The results show that TTA inhibits SW620 cell growth, at least partly, via the same mechanisms as DHA. Thus, cancer cells seem to cope with stress induced by various FAs through the same mechanisms.

## Methods

### Materials and cell culture

TTA was prepared at the Department of Chemistry, University of Bergen, Norway, as described previously [72] and in Supplementary experimental procedures (Additional file 2). All other solvents were of reagent grade from commercial sources. Antibody specifications are found in Additional file 2. The human colon carcinoma cell line SW620 was obtained from the American Type Culture Collection (ATCC, Rockville, MD) and cultivated in Leibovitz's L-15 medium (Cambrex, BioWhittaker, Walkersville, MD) supplemented with L-glutamine (2 mM), FBS (10%) and gentamicin (45 mg/l) (complete growth medium), in a humidified atmosphere of 5% CO_2_: 95% air at 37°C.

### Cell proliferation assay by xCELLigence

SW620 cells were seeded in 16 well plates (E-plate 16, Roche, Mannheim, Germany) (12.000 cells in 150 μl medium/well), following the xCELLigence Real Time Cell Analyzer (RTCA) DP instrument manual as provided by the manufacturer (Roche). After ~24 h, TTA (75 μM, prepared as described in Additional file 2) or the vehicle NaOH (control medium) was added, and the experiment was allowed to run for 72 h. Average baseline cell index and percent reduction of baseline cell index for TTA-treated cells compared to control cells were calculated for at least two measurements from 4 replicate experiments ± SD. Significance was calculated using one-tailed Student's t-test (P < 0.05).

### RNA isolation, gene expression profiling and statistical analysis

SW620 cells were seeded in 175 cm^2 ^flasks (3.5 × 10^6 ^cells in 28 ml medium). After 24 h incubation, cells were treated with TTA (75 μM) or NaOH (control) for 24 h. Cell harvesting and total RNA isolation was performed as elaborated in [28], using the High Pure RNA Isolation Kit (Roche). Gene expression profiling was performed following the Eukaryote expression manual from Affymetrix (Santa Clara, CA), using the Human Genome U133 2.0 Plus Array (Affymetrix), as described in Additional file 2. Statistical analysis of gene expression results was performed using the RMA method. Differentially expressed genes were identified using a linear model with a modified T-statistic [73], and results were corrected for multiple testing using the method described in [74] with a false discovery rate of 0.05. Annotations of probe sets and biological information of genes were retrieved from the web-based NetAffx Analysis Center at affymetrix.com and eGOn at genetools.no. Signal values presented are mean values of 3 replicate experiments. All experiments have been submitted to Array-Express with accession number E-MEXP-2590.

### Western blot analysis

Cells were treated with TTA (75 μM) or NaOH for 3, 6, 12, 24 and/or 48 h, as described in the gene expression profiling method. For detection of eIF2α-P and cyclin D1, cells were harvested as elaborated in [28], and cytosolic protein extracts were prepared immediately in lysis buffer as described in [63]. Nuclear protein extracts for detection of ATF4, CHOP, TRIB3 and C/EBPβ were obtained by using Nuclear Extract Kit (Active Motif, Rixensart, Belgium) according to the manufacturer's protocol. Protein concentrations were measured using Bio-Rad Protein Assay (Bio-Rad, Hercules, CA), and the protein extracts were snap-freezed in liquid nitrogen and stored at -80°C until further use. Cytosolic and nuclear proteins (80 or 50 μg per well, respectively) were separated on 10% precast denaturing NuPAGE^® ^gels (Invitrogen, Carlsbad, US) and transferred onto Immobilon polyvinylidene difluoride membranes (Millipore, Billerica, MA). Membranes were blocked in phosphate buffered saline buffer with 0.1% (v/v) Tween^® ^20 (BioRad, Hercules, CA) and 5% blocker non-fat dry milk, before incubated with primary and secondary horse radish peroxidase-conjugated antibodies (DAKO, Carpinteria, CA) dissolved in this blocking buffer as described in Additional file 2. eIF2α-P incubations were performed using tris-buffered saline Tween 20 buffer as described in [63]. Blots were detected by chemiluminescense using SuperSignal^® ^West Femto Maximum Sensitivity Substrate (Pierce, Rockford, IL) and visualized by Kodak Image Station 4000R (Eastman Kodak Co., Rochester, NY). Western blot band intensities were quantified using Kodak Molecular Imaging Software (version 4.0.1). Quantified western blot results, based on 3 independent replicates, were analysed as described in the proliferation assay method.

## List of abbreviations

Transcript short names are to be found in Table 1 and Additional file 1.

Cyt C: Cytochrome C; DHA: Docosahexaenoic acid; eIF2α: Eukaryote translation initiation factor 2 alpha; EIF2AK3/PERK: eIF2α kinase 3; eIF2α-P: phoshorylated eIF2α; EPA: Eicosapentaenoic acid; ER: Endoplasmic reticulum; ERAD: ER-associated degradation; FAs: Fatty acids; Hsps: Heat shock proteins; LAP: Liver enriched activator protein; LIP: Liver enriched inhibitor protein; n-3: Omega-3; PA: Palmitic acid; PUFAs: Polyunsaturated fatty acids; TTA: Tetradecylthioacetic acid; UPR: Unfolded protein response

## Competing interests

The authors declare that they have no competing interests. Rolf K Berge and Kjetil Berge have stocks in Thia Medica AS. There is no conflict of interest.

## Authors' contributions

AGL carried out cell experiments, xCELLigence experiments, western blots and helped to draft the manuscript. CHHP carried out cell experiments, gene expression experiments and drafted the manuscript. KB participated in study design, carried out initial experiments and helped to draft the manuscript. RKB participated in study design. SAS participated in study design and coordination and helped to draft the manuscript. All authors have read and approved the final manuscript.

## Supplementary Material

Additional file 1 **Supplementary gene expression results**. Functional categories of significantly differentially expressed transcripts affected in SW620 cells treated with TTA (75 μM) for 24 h, as determined by Affymetrix microarray analysis (P < 0.05). Click here for file

Additional file 2 **Supplementary experimental procedures**. The file contains supplementing information about the experimental procedures including preparation of TTA stock solution and medium, gene expression experiments and antibodies used. Click here for file
