# Supplementary material for: Tetradecylthioacetic acid inhibits proliferation of human SW620 colon cancer cells - gene expression profiling implies endoplasmic reticulum stress
Source: Lipids Health Dis. 2011 Oct 25;10:190. doi: 10.1186/1476-511X-10-190 (PMC3235040; doi:10.1186/1476-511X-10-190)
Supplement: Additional file 1 — Supplementary gene expression results. Functional categories of significantly differentially expressed transcripts affected in SW620 cells treated with TTA (75 μM) for 24 h, as determined by Affymetrix microarray analysis (P < 0.05). [file 1476-511X-10-190-S1.PDF]

Additional file 1

Title: Supplementary gene expression results

Description: Functional categories of significantly differentially expressed transcripts affected in SW620 cells treated with TTA (75 µM) for 24 h, as determined by Affymetrix microarray analysis ($P < 0.05$).

Gene Symbol	Affymetrix ID	Refseq NCBI ID	Transcript name	SW620 Fold Change 24h
**ER stress response**				
ATF3	202672_s_at	NM_001030287<br>NM_001040619<br>NM_001674<br>NM_004024	Activating transcription factor 3	3.5
ATF4	200779_at	NM_001675<br>NM_182810	Activating transcription factor 4	1.7
ATF6	217550_at<br>231927_at<br>203952_at	NM_007348	Activating transcription factor 6	1.5<br>1.3<br>1.2
CEBPB	212501_at	NM_005194	CCAAT/enhancer binding protein (C/EBP), beta	3.2
CHOP/DDIT3	209383_at	NM_001130101<br>NM_001130102<br>NM_004083<br>NM_005693	DNA-damage-inducible transcript 3	2.7
GADD34	37028_at<br>202014_at	NM_014330	Growth arrest and DNA-damage-inducible 34	2.0<br>2.0
IRE1/ERN1	235745_at	NM_001433	Endoplasmic reticulum to nucleus signaling 1	1.7
NRF2/NFE2L2	201146_at	NM_001145412<br>NM_001145413<br>NM_006164	Nuclear factor (erythroid-derived 2)-like 2	1.3
TRIB3	1555788_a_at<br>218145_at	NM_021158	Tribbles homolog 3 (Drosophila)	5.1<br>4.9
XBP1	242021_at	NM_001079539	X-box binding protein 1	1.2

	200670_at	NM_005080		1.6
**Amino acid transport and synthesis**				
AARS	201000_at	NM_001605	Alanyl-tRNA synthetase	1.7
ASNS	205047_s_at<br>241082_at	NM_001673<br>NM_133436<br>NM_183356	Asparagine synthetase	4.8<br>1.6
CARS	202402_s_at<br>212971_at<br>240982_s_at<br>240983_s_at	NM_001014437<br>NM_001014438<br>NM_001751<br>NM_139273	Cysteinyl-tRNA synthetase	1.8<br>1.8<br>1.5<br>1.7
CARS2	237555_at	NM_024537	Cysteinyl-tRNA synthetase 2, mitochondrial	1.3
EPRS	200841_s_at<br>200842_s_at<br>200843_s_at	NM_004446	Glutamyl-prolyl-tRNA synthetase	1.6<br>1.5<br>1.5
FARSB	232063_x_at	NM_005687	Phenylalanyl-tRNA synthetase, beta subunit	1.2
GARS	208693_s_at	NM_002047	Glycyl-tRNA synthetase	1.8
IARS	204744_s_at	NM_002161<br>NM_013417	Isoleucine-tRNA synthetase	1.4
LARS	217810_x_at<br>222427_s_at<br>222428_s_at	NM_020117	Leucyl-tRNA synthetase	1.3<br>1.2<br>1.3
MARS	201475_x_at<br>213671_s_at<br>213672_s_at	NM_004990	Methionine-tRNA synthetase	1.8<br>1.8<br>2.2
NARS	200027_at	NM_004539	Asparaginyl-tRNA synthetase	1.3
SARS	200802_at<br>237689_at	NM_006513	Seryl-tRNA synthetase	1.9<br>2.2
SLC1A4	209610_s_at<br>209611_s_at<br>212810_s_at<br>212811_x_at<br>244377_at	NM_003038<br>NM_001135581	Solute carrier family 1, member 4	4.1<br>1.7<br>2.9<br>3.1<br>2.0
SLC3A2	200924_s_at	NM_002394<br>NM_001012661<br>NM_001012662<br>NM_001012663<br>NM_001012664	Solute carrier family 3, member 2	1.4

		NM_001013251		
SLC6A9	207043_s_at	NM_001024845<br>NM_006934<br>NM_201649	Solute carrier family 6, member 9	1.6
SLC7A1	206566_at<br>212290_at<br>212292_at<br>212295_s_at	NM_003045	Solute carrier family 7, member 1	1.4<br>1.5<br>1.4<br>1.5
SLC7A11	209921_at<br>207528_s_at<br>217678_at	NM_014331	Solute carrier family 7, member 11	3.1<br>2.2<br>3.0
SLC38A1	218237_s_at<br>224579_at<br>224580_at	NM_001077484<br>NM_030674	Solute carrier family 38, member 1	1.3<br>1.2<br>1.3
SLC38A2	218041_x_at<br>220924_s_at<br>222982_x_at	NM_018976	Solute carrier family 38, member 2	1.7<br>1.7<br>1.6
SLC38A9	243709_at	NM_173514	Solute carrier family 38, member 9	1.5
SLC38A10	1563226_at<br>1563228_x_at	NM_001037984<br>NM_138570	Solute carrier family 38, member 10	1.2<br>1.3
SLC43A1	204394_at	NM_003627	Solute carrier family 43, member 1	1.3
TARS	240206_at	NM_152295	Threonyl-tRNA synthetase	1.8
WARS	200628_at<br>200629_at	NM_004184<br>NM_173701<br>NM_213645<br>NM_213646	Tryptophanyl-tRNA synthetase	1.7<br>1.7
YARS	212048_s_at<br>238760_at	NM_003680	Tyrosyl-tRNA synthetase	1.5<br>1.8
**Chaperones / Protein folding / Unfolded protein response/ ER stress associated protein degradation**				
CREB3L2	212345_s_at	NM_194071	cAMP responsive element binding protein 3-like 2	1.4
CREB3L3	234361_at	NM_032607	cAMP responsive element binding protein 3-like 3	3.0
DNAJA1	200880_at<br>200881_s_at	NM_001539	DnaJ (Hsp40) homolog, subfamily A, member 1	-1.5<br>-1.4
DNAJB1	200664_s_at<br>200666_s_at	NM_006145	DnaJ (Hsp40) homolog, subfamily B, member 1	-1.5<br>-1.4
DNAJB2	202500_at	NM_001039550<br>NM_006736	DnaJ (Hsp40) homolog, subfamily B, member B2	1.3

DNAJB14	222850_s_at	NM_001031723	DnaJ (Hsp40) homolog, subfamily B, member B14	1.2
DNAJC24	242562_at	NM_181706	DnaJ (Hsp40) homolog, subfamily C, member 24	1.4
EDEM1	203279_at	NM_014674	ER degradation enhancer, mannosidase alpha-like 1	1.3
EDEM3	220342_x_at	NM_025191	ER degradation enhancer, mannosidase alpha-like 3	1.2
ERO1LB	231944_at	NM_019891	ERO1-like beta (S. cerevisiae)	1.3
HMOX1/HSP32	203665_at	NM_002133	Heme oxygenase (decycling) 1	1.6
HSF4	210977_s_at	NM_001040667<br>NM_001538	Heat shock transcription factor 4	1.2
HSPA1A	200799_at	NM_005345	Heat shock 70kDa protein 1A	-1.2
HSPA13	202557_at<br>202558_s_at	NM_006948	Heat shock 70kDa protein 13	1.4<br>1.7
HYOU1	200825_s_at	NM_006389	Hypoxia up-regulated 1	1.3
PDIA2	206691_s_at	NM_006849	Protein disulfide isomerase family A, member 2	1.2
PFDN2	218336_at	NM_012394	Prefoldin 2	1.4
SEC61A1	217716_s_at	NM_013336	Sec61 alpha 1 subunit (S. cerevisiae)	1.2
SEC61A2	219499_at<br>228747_at	NM_001142627<br>NM_001142628<br>NM_018144	Sec61 alpha 2 subunit (S. cerevisiae)	1.3<br>1.2
SEC61B	244700_at	NM_006808	Sec61 beta subunit	1.6
SEC63	201914_s_at<br>201915_s_at<br>201916_s_at	NM_007214	SEC63 homolog (S. cerevisiae)	1.3<br>1.2<br>1.3
**Ubiquitine / Proteasome**				
CUL1	238509_at	NM_003592	Cullin 1	1.4
CUL4A	232466_at	NM_001008895<br>NM_003589	Cullin 4A	1.4
CUL7	36084_at<br>241747_s_at	NM_014780	Cullin 7	1.2<br>1.3
PSMA3	232648_at	NM_002788<br>NM_152132	Proteasome subunit, alpha type, 3	1.4
PSMB1	214289_at	NM_002793	Proteasome subunit, beta type, 1	1.3
PSMB7	244801_at	NM_002799	Proteasome subunit, beta type, 7	1.4
PSMD6	1555884_at	NM_014814	Proteasome 26S subunit, non ATPase, 6	1.3
PSMD7	238738_at<br>244515_at	NM_002811	Proteasome 26S subunit, non ATPase, 7	1.2<br>1.2

PSME3	200987_x_at<br>209853_s_at	NM_005789<br>NM_176863	Proteasome (prosome, macropain) activator subunit 3 (PA28 gamma; Ki)	-1.3<br>-1.3
PSME4	212222_at<br>237180_at	NM_014614	Proteasome (prosome, macropain) activator subunit 4	1.2<br>1.6
PSMG2	218467_at	NM_020232	Proteasome (prosome, macropain) assembly chaperone 2	-1.2
PSMG4	242055_at	NM_001128591<br>NM_001128592<br>NM_001135750	Proteasome (prosome, macropain) assembly chaperone 4	2.2
SIAH1	232365_at	NM_001006610<br>NM_003031	Seven in absentia homolog 1 (Drosophila)	1.4
SMURF1	212666_at	NM_020429<br>NM_181349	SMAD specific E3 ubiquitin protein ligase 1	1.3
SQSTM1	201471_s_at<br>213112_s_at<br>239004_at<br>244804_at	NM_001142298<br>NM_001142299<br>NM_003900	Sequestosome 1	2.1<br>3.1<br>1.3<br>2.1
UBE2B	239163_at	NM_003337	Ubiquitin-conjugating enzyme E2B (RAD6 homolog)	1.5
UBE2CBP	233327_at	NM_198920	Ubiquitin-conjugating enzyme E2C binding protein	1.2
UBE2D3	240383_at	NM_003340<br>NM_181886<br>NM_181887<br>NM_181888<br>NM_181889<br>NM_181890<br>NM_181891<br>NM_181892<br>NM_181893	Ubiquitin-conjugating enzyme E2D 3 (UBC4/5 homolog, yeast)	1.4
UBE2H	217799_x_at<br>221962_s_at<br>222420_s_at<br>222421_at<br>226681_at<br>227950_at	NM_003344<br>NM_182697	Ubiquitin-conjugating enzyme E2H (UBC8 homolog, yeast)	1.3<br>1.4<br>1.5<br>1.6<br>1.3<br>1.2
UBE2I	233360_at<br>1558088_a_at	NM_003345<br>NM_194259<br>NM_194260<br>NM_194261	Ubiquitin-conjugating enzyme E2I (UBC9 homolog, yeast)	1.4<br>1.4
UBE2Z	236107_at	NM_023079	Ubiquitin-conjugating enzyme E2Z	1.3

UBE2V1 / TMEM189	223186_at	NM_001032288<br>NM_001162505<br>NM_021988<br>NM_022442<br>NM_199129<br>NM_199144	Transmembrane protein 189 /// ubiquitin-conjugating enzyme E2 variant 1	1.2
UBE3B	232301_at	NM_130466<br>NM_183415	Ubiquitin protein ligase E3B	1.2
UBE3C	243519_at	NM_014671	Ubiquitin protein ligase E3C	1.2
UBR2	212756_s_at	NM_015255	Ubiquitin protein ligase E3 component n-recognin 2	1.2
UBR4	211950_at<br>215636_at	NM_020765	Ubiquitin protein ligase E3 component n-recognin 4	1.2<br>1.2
UBR5	1555888_at	NM_015902	Ubiquitin protein ligase E3 component n-recognin 5	1.3
UFM1	218050_at<br>242669_at	NM_016617	Ubiquitin-fold modifier 1	1.2<br>1.6
USP2	207211_at	NM_004205<br>NM_171997	Ubiquitin specific peptidase 2	1.2
USP3	221654_s_at	NM_006537	Ubiquitin specific peptidase 3	1.3
USP25	220419_s_at<br>223167_s_at<br>1555559_s_at	NM_013396	Ubiquitin specific peptidase 25	1.4<br>1.4<br>1.2
USP32	244871_s_at	NM_032582	Ubiquitin specific peptidase 32	1.2
USP34	207365_x_at<br>233595_at	NM_014709	Ubiquitin specific peptidase 34	1.5<br>1.5
USP53	237465_at	NM_019050	Ubiquitin specific peptidase 53	1.3
**$Ca2^{+}$ homeostasis, signaling and transport**				
ATP2B4/PMCA4	205410_s_at<br>212135_s_at<br>212136_at	NM_001001396<br>NM_001684	ATPase, Ca^{2+} transporting, plasma membrane 4	1.5<br>2.7<br>5.4
ATP2C2	214798_at	NM_014861	ATPase, Ca^{2+} transporting, type 2C, member 2	1.2
CALHM3	1554711_at	NM_001129742	Calcium homeostasis modulator 3	1.3
CALM1	200653_s_at<br>200655_s_at<br>209563_x_at<br>211984_at<br>211985_s_at<br>213688_at	NM_006888	Calmodulin 1 (phosphorylase kinase, delta)	-1.5<br>-1.5<br>-1.4<br>-1.7<br>-1.8<br>-1.9

CALM3	200623_s_at	NM_005184	Calmodulin 3 (phosphorylase kinase, delta)	-1.3
CALR	200935_at<br>212953_x_at<br>214315_x_at	NM_004343	Calreticulin	-2.2<br>-1.5<br>-1.5
CALU	238908_at	NM_001219	Calumenin	1.4
CAMK2D	225019_at	NM_001221<br>NM_172114<br>NM_172115<br>NM_172127<br>NM_172128<br>NM_172129	Calcium/calmodulin-dependent protein kinase II delta	1.3
CAMK2N1	218309_at	NM_018584	Calcium/calmodulin-dependent protein kinase II inhibitor 1	1.2
CAPN1	232012_at	NM_005186	Calpain 1, (mu/I) large subunit	1.4
CAPN2	208683_at<br>214888_at	NM_001146068<br>NM_001748	Calpain 2, large subunit	1.4<br>1.8
CAPN5	205166_at<br>226292_at	NM_004055	Calpain 5	1.4<br>1.2
CAPN9	210641_at	NM_006615<br>NM_016452	Calpain 9	-1.7
CAPN10	219333_s_at	NM_021251<br>NM_023083<br>NM_023085<br>NM_023089	Calpain 10	1.3
DGKG	242319_at	NM_001080744<br>NM_001080745<br>NM_001346	Diacylglycerol kinase, gamma 90kDa	1.3
HERPUD1	217168_s_at	NM_001010989<br>NM_001010990<br>NM_014685	Homocysteine-inducible, endoplasmic reticulum stress-inducible, ubiquitin-like domain member 1	1.9
ITPR1	244090_at	-----	Inositol 1,4,5-triphosphate receptor, type 1	1.3
ITPR2	202660_at<br>202662_s_at	NM_002223	Inositol 1,4,5-triphosphate receptor, type 2	-2.2<br>-1.4
ITPR3	201187_s_at<br>201188_s_at<br>201189_s_at	NM_002224	Inositol 1,4,5-triphosphate receptor, type 3	1.3<br>1.5<br>1.5
ITPRIP	225582_at	NM_033397	Inositol 1,4,5-triphosphate receptor interacting protein	1.2
ITPRIPL1	240037_at	NM_001008949	Inositol 1,4,5-triphosphate receptor interacting protein-like 1	-1.3

		NM_001163523<br>NM_001163524<br>NM_178495		
ITPRIPL2	227514_at<br>227792_at<br>1568619_s_at	NM_001034841	Inositol 1,4,5-triphosphate receptor interacting protein-like 2	1.6<br>1.4<br>1.5
PIK3C2A	213070_at<br>241905_at	NM_002645	Phosphoinositide-3-kinase, class 2, alpha polypeptide	1.2<br>1.7
PLCD3	234971_x_at<br>1552476_s_at	NM_133373	Phospholipase C, delta 3	1.3<br>1.3
PLCG1	216551_x_at	NM_002660<br>NM_182811	Pospholipase C, gamma 1	-1.2
S100A3	206027_at	NM_002960	S100 calcium binding protein A3	-1.3
S100A5	207763_at	NM_002962	S100 calcium binding protein A5	1.4
S100A6	217728_at<br>228923_at	NM_014624	S100 calcium binding protein A6	1.2<br>2.0
S100A10	200872_at<br>238909_at	NM_002966	S100 calcium binding protein A10	1.2<br>2.4
S100A11	200660_at<br>208540_x_at	NM_021039	S100 calcium binding protein A11 (calgizzarin)	1.5<br>1.5
S100A14	218677_at	NM_020672	S100 calcium binding protein A14	1.7
S100A16	227998_at	NM_080388	S100 calcium binding protein A16	1.2
S100P	204351_at	NM_005980	S100 calcium binding protein P	4.9
SLC3A2	200924_s_at	NM_001012661<br>NM_001012662<br>NM_001012663<br>NM_001012664<br>NM_001013251<br>NM_002394	Solute carrier family 3 (activators of dibasic and neutral amino acid transport), member 2	1.4
SLC11A2	203123_s_at<br>203124_s_at<br>203125_x_at<br>237106_at	NM_000617	Solute carrier family 11 (proton-coupled divalent metal ion transporters), member 2	1.4<br>1.2<br>1.2<br>1.4
SLC24A1	210421_s_at	NM_004727	Solute carrier family 24 (sodium/potassium/calcium exchanger), member 1	1.2
STC2	203438_at<br>203439_s_at	NM_003714	Stanniocalcin 2	2.1<br>2.7

**Cell cycle / Apoptosis**				
ATF5	204998_s_at<br>204999_s_at	NM_012068	Activating transcription factor 5	1.5<br>1.7
AURKA	208080_at	NM_003600<br>NM_198433<br>NM_198434<br>NM_198435<br>NM_198436<br>NM_198437	Aurora kinase A	-1.3
BIRC5	202094_at<br>202095_s_at<br>210334_x_at	NM_001012270<br>NM_001012271<br>NM_001168	Baculoviral IAP repeat-containing 5 / Survivin	-1.7<br>-1.3<br>-1.3
BRCA1	204531_s_at<br>211851_x_at	NM_007294<br>NM_007295<br>NM_007296<br>NM_007297<br>NM_007298<br>NM_007299<br>NM_007300<br>NM_007302<br>NM_007303<br>NM_007304<br>NM_007305	Breast cancer 1, early onset	-1.2<br>-1.2
BRCA2	208368_s_at<br>214727_at	NM_000059	Breast cancer 2, early onset	-1.4<br>-1.4
CASP4	213596_at	NM_001225<br>NM_033306	Caspase 4	1.4
CASP10	205467_at	NM_001230<br>NM_032974<br>NM_032977	Caspase 10, apoptosis-related cysteine peptidase	1.2
CCNA2	203418_at<br>213226_at	NM_001237	Cyclin A2	-1.2<br>-1.2
CCND3	201700_at	NM_001136017<br>NM_001136125<br>NM_001136126<br>NM_001760	Cyclin D3	-2.0
CCNE1	213523_at	NM_001238<br>NM_057182	Cyclin E1	-1.3

CCNE2	205034_at<br>211814_s_at	NM_057749	Cyclin E2	-2.9<br>-3.1
CCNF	204827_s_at	NM_001761	Cyclin F	-1.2
CDC2	203214_x_at	NM_001130829<br>NM_001786<br>NM_033379	Cell division cycle 2, G1 to S and G2 to M	-1.2
CDC2L1 ///<br>CDC2L2	215329_s_at	NM_024011<br>NM_033486<br>NM_033487<br>NM_033488<br>NM_033489<br>NM_033492<br>NM_033493<br>NM_033529	Cell division cycle 2-like 1 (PITSLRE proteins) /// cell division cycle 2-like 2 (PITSLRE proteins)	1.2
CDC2L2	212401_s_at	NM_024011<br>NM_033529	Cell division cycle 2-like 2 (PITSLRE proteins)	1.2
CDC5L	209055_s_at<br>209056_s_at	NM_001253	CDC5 cell division cycle 5-like (S. pombe)	-1.3<br>-1.2
CDC6	203967_at<br>203968_s_at	NM_001254	Cell division cycle 6 homolog (S. cerevisiae)	-1.9<br>-1.9
CDC7	204510_at	NM_001134419<br>NM_001134420<br>NM_003503	Cell division cycle 7 homolog (S. cerevisiae)	-1.2
CDC14A	205288_at<br>210742_at	NM_003672<br>NM_033312<br>NM_033313	CDC14 cell division cycle 14 homolog A (S. cerevisiae)	-1.3<br>1.2
CDC16	209658_at<br>209659_s_at	NM_001078645<br>NM_003903	Cell division cycle 16 homolog (S. cerevisiae)	1.2<br>1.2
CDC20	202870_s_at	NM_001255	Cell division cycle 20 homolog (S. cerevisiae)	1.2
CDC23	202892_at<br>223651_x_at	NM_004661	Cell division cycle 23 homolog (S. cerevisiae)	-1.2<br>-1.3
CD24	266_s_at<br>208651_x_at<br>209771_x_at<br>216379_x_at	NM_004235	CD24 molecule	-1.6<br>-1.3<br>-1.5<br>-1.5
CDC25A	204695_at<br>204696_s_at<br>1555772_a_at	NM_001789<br>NM_201567	Cell division cycle 25 homolog A (S. pombe)	-1.6<br>-1.3<br>-1.9

CDC26	225422_at	NM_139286	Cell division cycle 26 homolog (S. cerevisiae)	-1.4
CDC27	217879_at<br>217880_at<br>217881_s_at	NM_001114091<br>NM_001256	Cell division cycle 27 homolog (S. cerevisiae)	-1.2<br>-1.3<br>-1.2
CDC45L	204126_s_at	NM_003504	CDC45 cell division cycle 45-like (S. cerevisiae)	-1.4
CDC73	218578_at	NM_024529	Cell division cycle 73, Paf1/RNA polymerase II complex component, homolog (S. cerevisiae)	-1.4
CDC123	201725_at	NM_006023	Cell division cycle 123 homolog (S. cerevisiae)	-1.3
CDCA5	224753_at	NM_080668	Cell division cycle associated 5	-1.5
CDK2	204252_at<br>211804_s_at	NM_001798<br>NM_052827	Cyclin-dependent kinase 2	-1.3<br>-1.4
CDK2AP1	201938_at	NM_004642	Cyclin-dependent kinase 2 associated protein 1	-1.4
CDK3	207188_at<br>229468_at	NM_001258	Cyclin-dependent kinase 3	1.3<br>1.3
CDK4	202246_s_at	NM_000075	Cyclin-dependent kinase 4	-1.4
CDK5	204247_s_at	NM_001164410<br>NM_004935	Cyclin-dependent kinase 5	-1.5
CDK5R1	204995_at	NM_003885	Cyclin-dependent kinase 5, regulatory subunit 1 (p35)	-1.8
CDK6	224847_at<br>224848_at<br>224851_at<br>243000_at	NM_001145306<br>NM_001259	Cyclin-dependent kinase 6	-1.4<br>-1.5<br>-1.3<br>-1.3
CDK7	211297_s_at	NM_001799	Cyclin-dependent kinase 7	1.2
CDKN1C	213182_x_at<br>213348_at	NM_000076<br>NM_001122630<br>NM_001122631	Cyclin-dependent kinase inhibitor 1C (p57, Kip2)	-1.2<br>-1.4
CDKN2A	211156_at	NM_000077<br>NM_058195<br>NM_058197	Cyclin-dependent kinase inhibitor 2A (melanoma, p16, inhibits CDK4)	1.3
CDKN2B	207530_s_at<br>236313_at	NM_004936<br>NM_078487	Cyclin-dependent kinase inhibitor 2B (p15, inhibits CDK4)	1.5<br>12.0
CDKN2C	204159_at<br>211792_s_at	NM_001262<br>NM_078626	Cyclin-dependent kinase inhibitor 2C (p18, inhibits CDK4)	-1.8<br>-1.3
CDKN2D	210240_s_at	NM_001800<br>NM_079421	Cyclin-dependent kinase inhibitor 2D (p19, inhibits CDK4)	1.2
CDKN3	209714_s_at<br>1555758_a_at	NM_001130851<br>NM_005192	Cyclin-dependent kinase inhibitor 3	-1.4<br>-1.3

CEBPB	212501_at	NM_005194	CCAAT/enhancer binding protein (C/EBP), beta	3.2
CINP	218267_at	NM_032630	Cyclin-dependent kinase 2 interacting protein	-1.3
CUL1	238509_at	NM_003592	Cullin 1	1.4
DUSP1	201041_s_at<br>201044_x_at	NM_004417	Dual specificity phosphatase 1	1.7<br>1.4
GADD34	37028_at<br>202014_at	NM_014330	Growth arrest and DNA-damage-inducible 34	2.0<br>2.0
GADD45A	203725_at	NM_001924	Growth arrest and DNA-damage-inducible, alpha	1.4
HMOX1 / HSP32	203665_at	NM_002133	Heme oxygenase (decycling) 1	1.6
KLF4	220266_s_at<br>221841_s_at	NM_004235	Kruppel-like factor 4 (gut)	1.9<br>2.4
KLF11	218486_at<br>1553137_s_at	NM_003597	Kruppel-like factor 11	1.9<br>1.4
PCNA	201202_at<br>217400_at	NM_002592<br>NM_182649	Proliferating cell nuclear antigen	-1.7<br>-1.4
PDCD2	213581_at	NM_002598<br>NM_144781	Programmed cell death 2	-1.4
PDCD4	202731_at<br>212593_s_at	NM_014456<br>NM_145341	Programmed cell death 4 (neoplastic transformation inhibitor)	1.3<br>1.3
PDCD6	222152_at<br>222380_s_at	NM_013232	Programmed cell death 6	1.3<br>1.3
PLK2	201939_at	NM_006622	Polo-like kinase 2 (Drosophila)	-1.3
POLA1	204835_at	NM_016937	Polymerase (DNA directed), alpha 1, catalytic subunit	-1.5
POLD1	203422_at	NM_002691	Polymerase (DNA directed), delta 1, catalytic subunit 125kDa	-1.3
POLE	216026_s_at	NM_006231	Polymerase (DNA directed), epsilon	-1.3
PPARD	37152_at<br>208044_s_at<br>242218_at	NM_006238<br>NM_177435	Peroxisome proliferator-activated receptor delta	1.5<br>1.4<br>1.3
SFN	33322_i_at<br>33323_r_at<br>209260_at	NM_006142	Stratifin	1.6<br>1.7<br>1.6
VEGFA	210512_s_at<br>210513_s_at<br>211527_x_at<br>212171_x_at	NM_001025366<br>NM_001025367<br>NM_001025368<br>NM_001025369<br>NM_001025370<br>NM_001033756	Vascular endothelial growth factor	3.6<br>2.3<br>3.3<br>2.8

		NM_003376		
**Assorted**				
TAGLN	205547_s_at<br>1555724_s_at	NM_001001522<br>NM_003186	Transgelin	3.4<br>2.5
EREG	205767_at<br>1569583_at	NM_001432	Epiregulin	2.0<br>2.6
JAG1	209097_s_at<br>209098_s_at<br>209099_x_at<br>216268_s_at<br>231183_s_at	NM_000214	Jagged 1 (Alagille syndrome)	1.6<br>3.5<br>2.7<br>2.9<br>2.4
AKAP12	210517_s_at<br>227529_s_at<br>227530_at	NM_005100<br>NM_144497	A kinase (PRKA) anchor protein 12	3.2<br>3.4<br>3.5
ADRP/PLIN2	209122_at	NM_001122	Perilipin 2	2.8
