# Supplementary material for: Tetradecylthioacetic acid inhibits proliferation of human SW620 colon cancer cells - gene expression profiling implies endoplasmic reticulum stress
Source: Lipids Health Dis. 2011 Oct 25;10:190. doi: 10.1186/1476-511X-10-190 (PMC3235040; doi:10.1186/1476-511X-10-190)
Supplement: Additional file 2 — Supplementary experimental procedures. The file contains supplementing information about the experimental procedures including preparation of TTA stock solution and medium, gene expression experiments and antibodies used. [file 1476-511X-10-190-S2.PDF]

Additional file 2

Title: Supplementary experimental procedures.

Description: The file contains supplementing information about the experimental procedures including preparation of TTA stock solution and medium, gene expression experiments and antibodies used.

Preparation of TTA stock solution and medium

TTA powder was prepared at the Department of Chemistry, University of Bergen, Norway, as described in [72]. Stock solution of TTA (25 mM TTA in 0,1 M NaOH) was made by dissolving TTA in NaOH at 80°C and stored at -20°C. Before experiments, TTA stock solution was heated to 80°C, dissolved and complexed in warm FBS (37°C), before dilution in complete growth medium to a concentration of 75 µM.

Gene expression experiments

Microarray experiments were performed according to the eukaryote expression manual (Affymetrix, Santa Clara, CA) using standard chemicals from Affymetrix. 5 µg total RNA was used for double-stranded cDNA synthesis using the Poly-A RNA Control Kit and One-Cycle cDNA synthesis Kit. cRNA was synthesized using the IVT Labeling Kit. Both cDNA and cRNA were cleaned up using Sample Clean Up Module. 15 µg fragmented cRNA were hybridized to the Human Genome U133 Plus 2.0 Array using the Hybridization, Wash and Stain Kit and Hybridization Control Kit. Hybridization and washing/staining were performed using Hybridization Oven 640 and Fluidics Station 450. Arrays were scanned on an Affymetrix GeneChip 3000 7G Scanner controlled by Affymetrix GeneChip Command Console (AGCC).

Antibodies

Western blot membranes were probed (at 4 °C over night) with primary antibodies detecting the following proteins; Phospho-eIF2α (Ser 51, CST-9721-S, rabbit polyclonal antibody, Cell Signalling Technology, Danvers, MA), Cyclin D1 (2926, mouse monoclonal antibody, Cell Signalling Technology), CREB-2 (C-20/ATF4, sc-200, rabbit polyclonal antibody, Santa Cruz Biotechnology, CA), CHOP (MA1-250, mouse monoclonal antibody, Affinity BioReagents, Golden, CO), TRIB3 (ab73547, rabbit polyclonal antibody, Abcam, Cambridge, UK) and C/EBP β (sc-150, C-19, rabbit polyclonal antibody, Santa Cruz Biotechnology). Phospho-eIF2α probed membranes were stripped in Restore Western blot Stripping Buffer (Pierce, Rockford, IL) and reprobed with total eIF2α (CST-9722, rabbit polyclonal antibody, Cell Signalling Technology) as a control. Membranes probed with cytosolic and nuclear protein extracts were reprobed with beta Actin (AC-15, AB6276, mouse monoclonal antibody, Abcam) and Lamin A/C (sc-6215, goat polyclonal antibody, Santa Cruz Biotechnology), respectively, as loading controls. All membranes were probed (one hour at room temperature) with horseradish peroxidise (HRP)-conjugated secondary antibodies (anti-rabbit, anti-mouse or anti-goat polyclonal antibodies, DAKO, Carpinteria, CA).
